# Long-Life and pH-Stable SnO_2_-Coated Au Nanoparticles for SHINERS

**DOI:** 10.1021/acs.jpcc.2c02432

**Published:** 2022-07-13

**Authors:** Julia Fernández-Vidal, Ana M. Gómez-Marín, Leanne A. H. Jones, Chih-Han Yen, Tim D. Veal, Vinod R. Dhanak, Chi-Chang Hu, Laurence J. Hardwick

**Affiliations:** † Stephenson Institute for Renewable Energy, Department of Chemistry, Peach Street, University of Liverpool, Liverpool L69 7ZF, United Kingdom; ‡ Department of Chemistry, Division of Fundamental Sciences (IEF) Aeronautics Institute of Technology (ITA) Praça Marechal Eduardo Gomes, 50 CEP 12228-900 São José dos Campos, São Paulo, Brazil; § Stephenson Institute for Renewable Energy and Department of Physics, Peach Street, University of Liverpool, Liverpool L69 7ZF, United Kingdom; ∥ Department of Chemical Engineering, National Tsing Hua University, Hsinchu 300044, Taiwan

## Abstract

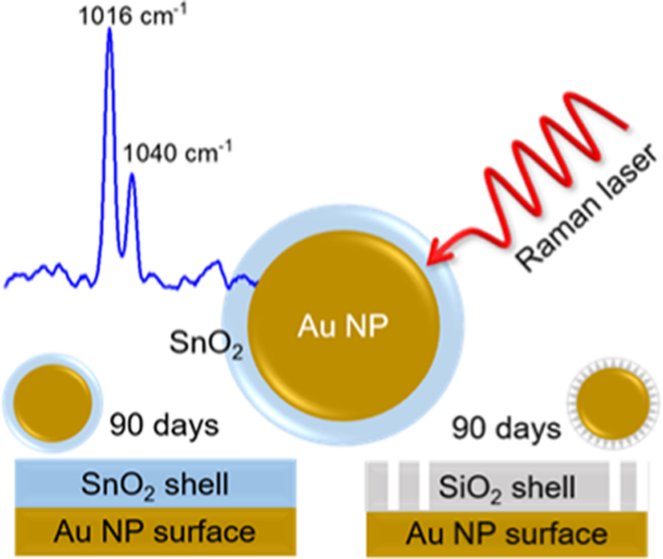

Shell-isolated nanoparticles (SHINs) with a 37 nm gold core and an 11 nm tin dioxide (SnO_2_) coating exhibited long-life Raman enhancement for 3 months and a wide pH stability of pH 2–13 in comparison with conventional SiO_2_-coated SHINs. Herein, Au–SnO_2_ is demonstrated as a more durable SHIN for use in the technique Shell-Isolated Nanoparticles for Enhanced Raman Spectroscopy (SHINERS).

## Introduction

Surface-enhanced Raman spectroscopy (SERS) is a powerful technique that merges the intrinsic features of Raman spectroscopy^1^ with the high sensitivity and enhancement that allows the detection of single molecules^2,3^ and is widely used in many fields^4^ due to its high surface sensitivity and applicability.^5,6^ In SERS, the enhanced Raman scattering comes mainly from the interaction of the plasmon band of metals, in the form of either roughened surfaces or plasmonic nanoparticles, with the adsorbed molecules.^5,7,8^ Nonetheless, SERS studies were generally limited to gold, silver, and copper roughened surfaces^9^ until 2010, when Li et al.^10^ and co-workers developed shell-isolated nanoparticles for enhanced Raman spectroscopy (SHINERS). SHINERS consists of the use of shell-isolated nanoparticles (SHINs) with a core made of metals with surface plasmon resonance (SPR) properties in the visible region (usually gold, due to a straightforward synthesis route) covered with an ultrathin (1–4 nm), chemically inert shell.^10^ In SHINERS, the plasmonic core of SHINs induces local enhancements in the intensity of the electric field and hence, an increase of the Raman signal,^11,12^ while the shell isolates the nucleus, eliminating any chemical or electrical interaction between the metallic core and any other parts of the system.^13^ This isolation of the plasmonic probe makes SHINERS a key technique to overcome the limitations of SERS, where the properties of the bare metal may interfere with the measurements, and to investigate electrified interfaces in operando conditions, giving high-quality Raman spectra of adsorbates on different surfaces that have formerly been difficult to analyze.^14,15^

Silicon dioxide (SiO_2_) is the most used shell material for SHINs.^14−18^ It is optically and chemically inert, and it readily forms a thin, uniform, pinhole-free shell that permits the core of SHINs to be isolated. However, one of the challenges for developing SiO_2_-coated particles applicable for SHINERS is to cover the plasmonic core with a suitable shell that fulfills specific characteristics for this application. In general, the shell must be 1–4 nm thick, pinhole-free, nonporous, and electrochemically inert (for most applications), to both isolate the core and allow a large enhancement factor (EF)^19−22^ of the Raman signal to occur. SiO_2_ shells thicker than 4 nm reduce or even block the enhancement,^15^ and the synthesis of a thinner shell is more complex and generally results in the formation of pinholes. On the other hand, SiO_2_ shells also present some limitations for use in catalysis and electrochemistry due to their narrow pH stability (pH 7–11.5),^6,23^ short shelf life (approximately 2 weeks), and stability when used at elevated temperatures above 30 °C. Therefore, the development of new shell materials to cover metals with SPR properties in the visible region is currently an active field.^6,9,13,23,24^

Alternative oxide coatings that have been reported include Al_2_O_3_, SnO_2_, MnO_2_, TiO_2_, or ZrO_2_.^14^ Al_2_O_3_ shells have proven to give similar results to SiO_2_ coatings with similar shell thicknesses; however, they also dissolve at alkaline pH.^10^ Ultrathin MnO_2_ shells^6^ obtained by reducing KMnO_4_ using K_2_C_2_O_4_ have been demonstrated to achieve greater Raman enhancement and pH stability in alkaline media than SiO_2_ and Al_2_O_3_ shells.^6^ TiO_2_ coatings obtained through the hydrolysis of titanium tetraisopropoxide^25,26^ have also been used in catalysis and shown to be highly thermally stable (up to 400 °C).^27^ Krajczewski et al.^28^ developed ZrO_2_ shells by decomposing zirconium (IV) propoxide for use in SHINERS, which had previously been shown to provide a high pH stability in Ag-ZrO_2_ nanoparticles.^29^ Tin(IV) dioxide (SnO_2_) is also another promising shell material.^30,31^ It does not dissolve at highly acidic and basic pH,^23,32^ remains stable over a wide pH range of ca. pH 0 to 12 (from the Pourbaix diagram),^33^ and is also transparent^34^ in the visible range of the light spectrum, making it a practical material for optical coatings. Indeed, Burgess and co-workers reported on SnO_2_-Au SHINs to study steel corrosion under alkaline conditions.^32^ SnO_2_ coatings have been demonstrated to protect metals from corrosion for cathodic protection applications,^35^ opening up the possibility of using SnO_2_ as a shell material to coat metallic cores like Ag or Au@Ag nanoparticles that, besides presenting a higher SERS activity than Au nanoparticles, are vulnerable to oxidation.^36^

Additionally, it has been reported that SnO_2_ octahedral nanoparticles alone are SERS active,^37^ ascribed to a charge-transfer (CT) mechanism that in turn may contribute to the signal enhancement of the metal core,^38^ opening the possibility of utilizing thicker shell coatings. Moreover, because SnO_2_ does not dissolve in fluoride-containing solutions, as in the case of SiO_2_, SHINs with a SnO_2_ shell would offer the possibility of exploring the electrochemical interface in the neutral-pH region in buffered NaF/HF solutions, avoiding local pH changes while maintaining the absence of specifically adsorbed anions.

In this paper, SHINs with SnO_2_ shells are shown to be more durable, applicable in a wider pH range, and a robust alternative to the commonly used SiO_2_, confirming their suitability for SHINERS.

## Methods

All of the glassware used throughout was washed with aqua regia (3HCl:1HNO_3_) and rinsed with distilled water (Milli-Q, 19 MΩ) several times until fully cleaned. Persistent stains on the glass walls were removed using piranha solution (3H_2_SO_4_:1H_2_O_2_).

### Synthesis of Citrate-Stabilized Gold Nanoparticles

The synthesis of nanoparticles was carried out using the Turkevich-Frens citrate reduction method.^39−42^ In a conical flask, 2.43 mL of 0.83% HAuCl_4_ (99.995%, Sigma-Aldrich) was diluted with 200 mL of distilled water (Milli-Q, 19 MΩ). The resulting solution was heated under vigorous stirring until boiling. Immediately, 1.5 mL of 1% citric acid (≥99.5%, Sigma-Aldrich) was added to the solution of HAuCl_4_. After a few seconds, the solution changed from a pale-yellow color to transparent, then black, and finally maroon. The suspension of nanoparticles was stirred for another 20 min and then allowed to cool overnight at room temperature. The obtained nanoparticle suspensions were stored away from light sources for the duration of the experiments, remaining stable throughout the process.

### Synthesis of SnO_2_-Coated SHINs

The coating of the gold nanoparticles was carried out by the precipitation method described by Mulvaney et al.^31^ with slight modifications. Details of the coating of the nanoparticles are as follows. Twenty-five milliliters of the gold nanoparticles (∼0.226 nM) was diluted in 50 mL of distilled water (Milli-Q, 19 MΩ), placed in a round-bottom flask under strong stirring, and heated to 60 °C for 20 min in a hot water bath. The pH of the solution was adjusted to 11.4 with 1 M NaOH (≥98%, Sigma-Aldrich) to permit the precipitation of SnO_2_. Since bigger nanoparticles were used for this synthesis (37 vs the 15 nm NPs from the Mulvaney et al. protocol),^31^ a higher pH (11.4) was set to ensure that the pH will remain over 10.5 once the precursor was added. Different volumes of 0.2 M sodium stannate trihydrate (Na_2_SnO_3_·3H_2_O) (95%, Sigma-Aldrich) were added quickly to achieve nucleation of SnO_2_ on the surface of the gold nanoparticles. The solution was kept at 60 °C until the shell reached the desired thickness. To check the effect of temperature and pH on the shell formation process, three synthesis procedures were performed with the following details: (1) following the previous description at 60 °C, (2) at 80 °C, and (3) following the above description but skipping the pH adjustment step, giving rise to a final solution pH of 3.5.

### Characterization

Raman measurements were carried out with a Raman microscope (Renishaw InVia), using a 785 nm laser with an exposure time of 10 sec. Raman spectra were calibrated vs the 520 cm^–1^ peak of Si with a resolution of 1.1 cm^–1^. For Raman pinhole tests, 5 μL of SnO_2_-coated SHINs (∼1.13 nM) was deposited onto a silicon wafer (Si(100), Agar Scientific) by drop casting. Then, 2 μL of a 10 mM pyridine (99.8%, Sigma-Aldrich) solution was dropped on top of the deposited NPs. Enhancement tests were similarly performed, but using a gold film on silicon substrates (Au(111), Platypus Technologies).

The structure, surface morphology, average particle of the SHINs, and shell thicknesses were measured by transmission electron microscopy (TEM). Images were recorded using a JEOL 2100F Cs-corrected microscope operated at 200 kV. Shell thicknesses, and nanoparticle sizes were determined using the software Gwyddion (Version 2.55). Samples were prepared from a dilute colloidal dispersion of synthesized nanoparticles (∼0.226 nM), by evaporating ca. 10 μL on Lacey Carbon Films on 300 Mesh Copper Grids (Agar Scientific). In all, 20–50 nanoparticles were measured in each case to obtain the shell thicknesses and nanoparticles sizes’ mean and standard deviation values.

To determine the possible influence of the pH during the synthesis on the chemical properties of the SnO*_x_* shell, X-ray photoelectron spectroscopy (XPS) was used to study the oxidation state of SnO_x_. Measurements were performed using a Thermo Fisher Scientific NEXSA spectrometer with a micro-focused monochromatic Al K_α_ source (1486.6 eV). Low-energy electrons and Ar ions were used for charge neutralization. Photoelectrons were measured at a pass energy of 40 eV for core levels and 200 eV for survey spectra, with an energy step size of 0.1 and 1 eV, respectively. These measurements were performed at a base pressure of 5 × 10^–8^ mbar. XPS measurements were repeated using a different spectrometer and were found to be consistent. Measurements from the second spectrometer are shown here. The spectrometer consists of a SPECS monochromatic Al K_α_ x-ray source (1486.6 eV) operating at 250 W and a hemispherical PSP Vacuum Technology electron-energy analyzer operating with a pass energy of ∼10 eV. Calibration was performed using a silver sample, enabling the energy resolution of 0.4 eV to be determined by fitting the Fermi edge data. The base pressure of this system was 1 x 10^-10^ mbar. Samples were prepared from 10 μL of the core/shell nanoparticles dispersion (∼1.13 nM) deposited and evaporated at room temperature until fully dry in silicon wafer (Si(100), Agar Scientific). To form a thick layer, the deposition was repeated 3–6 times to achieve greater coverage of the substrate. Peaks were simulated with combined profiles of Gaussian-Lorentzian functions, and a Shirley background was used to model the inelastic scattering of photoelectrons. The accuracy of the measurements was ±0.1 eV. In the analysis of Sn 3d XPS spectra, Sn^2+^ and Sn^4+^ oxidation states were considered. Sn 3d_5/2_ and Sn 3d_3/2_ peaks, separated by 8.4 eV, with a 3:2 peak intensity ratio, and equal full width at half maximum (FWHM) values, were represented by GL (30) functions. High-resolution spectra for O 1s were also analyzed.

Powder X-ray diffraction (PXRD) patterns were collected, and crystallite size was calculated for Au–SnO_2_ SHINs. 2θ patterns were collected in the angular range 20° ≤ θ ≤ 90° in steps of 0.01° using a Rigaku SmartLab diffractometer. Samples were prepared from 10 μL of the core/shell nanoparticles dispersion (∼1.13 nM) deposited and evaporated at room temperature until fully dry in plain 0.8–1.0 mm thick glass slides. To form a thick layer, the deposition was repeated 3–6 times to achieve greater coverage of the substrate. Crystallite sizes of Au and SnO_2_ were calculated using Scherrer equation^43,44^ (eq 1).
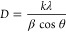
1where *D* is the crystallite size, *k* is the Scherrer constant depending on the crystallite shape, λ is the wavelength of the X-ray source (1.541 Å for Cu), β is the FWHM in radians, and *θ*is the Bragg angle corresponding to the peak position in radians. Three main peaks for the SnO_2_ shell (JCPDS 41–1445), corresponding to the (110), (101), and (211) planes were used to calculate the crystallite sizes. Correspondingly, crystallite size calculations for Au nanoparticles (JCPDS 89–3697) were performed by employing peaks relative to the (111), (200), and (220) planes. The reported crystallite sizes correspond to the average values between those calculations.

## Results and Discussion

### Characterization of SnO_2_ shell and synthesis

**Figure 1:**
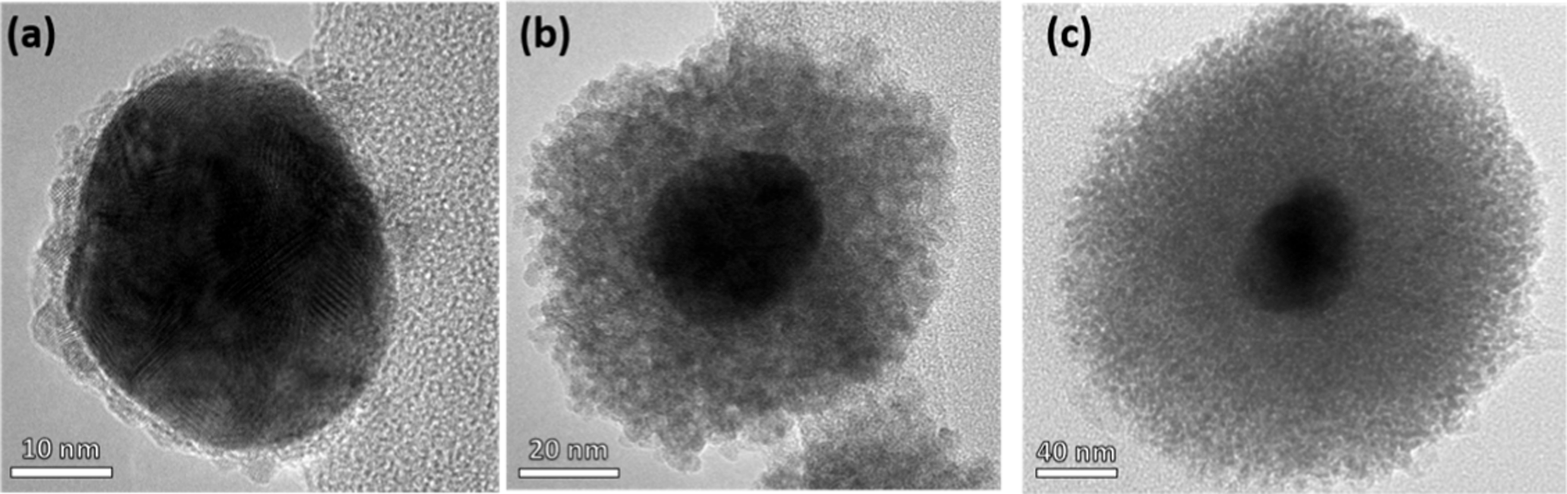
SnO_2_-coated Au NPs at pH 3.5 and 60 °C (a), pH 11.4 and 60 °C (b), and pH 11.4 and 80 °C (c) after 240 min of reaction time.

Tin dioxide (SnO_2_)-coated nanoparticles were synthesized by the hydrothermal method given in the Experimental section and described in the literature^23,31^ using 37 ± 5 nm Au NPs (See Figure S1 in Supporting Information) as nuclei for the SHINs. To study the effect of pH and temperature in the formation of SnO_2_-coated Au NPs and optimize the synthesis of SHINs, SnO_2_ shells were obtained at pH 3.5 at 60 °C, pH 11.4 at 60 °C, and pH 11.4 at 80 °C and samples were taken at different reaction times. The TEM results in Figure 1 show images of the coated NPs after 240 min reaction time, and the characteristics of the syntheses are displayed in Table 1.

**Table 1:** Experimental Details, Surface Plasmon (SP) Band Position, and Shell Thicknesses from the TEM Images Represented in Figure 1

pH	3.5	11.4	11.4
temperature	60 °C	60 °C	80 °C
time reaction	240 min	240 min	240 min
Na_2_SnO_3_ concentration	15 mM	8.04 mM	1.81 mM
SP band position	542 nm	565 nm	565 nm
shell thickness	2.6 ± 1.2 nm	23.7 ± 3 nm	53.7 ± 5.8 nm

A different shell morphology for all three different conditions is observed in Figure 1. The slow growth of a thin shell on the samples at pH 3.5 suggests a pH-dependent formation of SnO_2_ consistent with previously reported protocols^31^ for core/shell SnO_2_ particles where an alkaline pH is crucial for SnO_2_ precipitation. At pH 11.4, thicker, more porous shells are observed at 80 °C due to a faster reaction kinetics at this temperature compared to 60 °C.

It has been reported^45−47^ that the position of the absorption band is directly related to the shell thickness. Thicker shells exhibit increased scattering and have larger optical cross sections and red-shifted bands. However, the absorption band also depends on the refractive index and density of the shell.^31,47,48^ In this framework, it would be expected that more porous shells have a lower density, and a lower shift on the absorbance peak compared to citrate-stabilized Au NPs. This is supported by the TEM images of the SnO_2_-coated Au NPs (Figure 1). Despite the samples at pH 11.4 presenting the same absorption band position (∼565 nm) (Figure S3), neither the thickness of the shell (Table 1) nor the granular texture under TEM agrees, proving UV–visible measurements alone to be an unsuitable method to characterize the shell thickness of SnO_2_-Au SHINs.

Only those SnO_2_-coated nanoparticles whose coating was synthesized at 60 °C did not present any pinholes (Figures S4 and S5), implying that the synthesis conditions are the key to achieve suitable SHINERS. A high-intensity enhancement is detected in the pinhole-free samples after a reaction time of 80 min and at an Na_2_SnO_3_ concentration of 8.04 mM. This enhancement diminishes as the reaction time increases, suggesting an enlargement in shell thickness consistent with TEM images (Figure S3). SHINs with thinner shells obtained at pH 3.5 present a larger enhancement of the Raman signal as the distance between the plasmonic nanoparticles is shorter;^49^ however, pinholes are detected in those cases (Figure S4). The porosity of the shell could explain the presence of pinholes even in SHINs with the thickest coatings at 80 °C.^50^

### Surface Analysis of SHINs

Differences in shell thickness because of pH media may occur due to the amphoteric nature of SnO_2_ in aqueous solutions,^51^ with a point of zero charge (pzc) around either pH_pzc_ 4.3^31^ or 6.05.^52^ Regardless of the exact value, a positive surface charge is expected at a pH <pH_pzc_, affecting the reactions taking place at the SnO_2_ surface.^52^ Considering the formation of the SnO_2_ shell by eq 2,^51^ the positively charged surface of SnO_2_ at pH 3.5 would be responsible for the slower shell growth under these conditions, due to the surface electrostatic repulsion of Sn^4+^ cations.
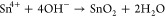
2The XPS signals of Na 1s and Na 2s in the survey spectra and a feature at 497.5 eV for the Sn 3d spectrum corresponding to the Na KLL Auger line are also observed at pH 3.5 (Figures S6 and S7) from the Na_2_SnO_3_ precursor used in the synthesis. Changes in the surface charge have been explained^53,54^ according to eqs 3 and 4. An alternative proposed mechanism includes the metal reduction accompanied by the simultaneous proton intercalation into the oxide,^54^ similar to eq 5. In any case, it could be possible that instead of H^+^, a Na^+^ cation from the solution would be adsorbed/intercalated into the SnO_2_ surface, which could explain the XPS signal from Na^+^ cations observed in Figures S6 and S7.
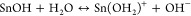
3
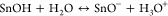
4
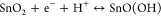
5

**Figure 2:**
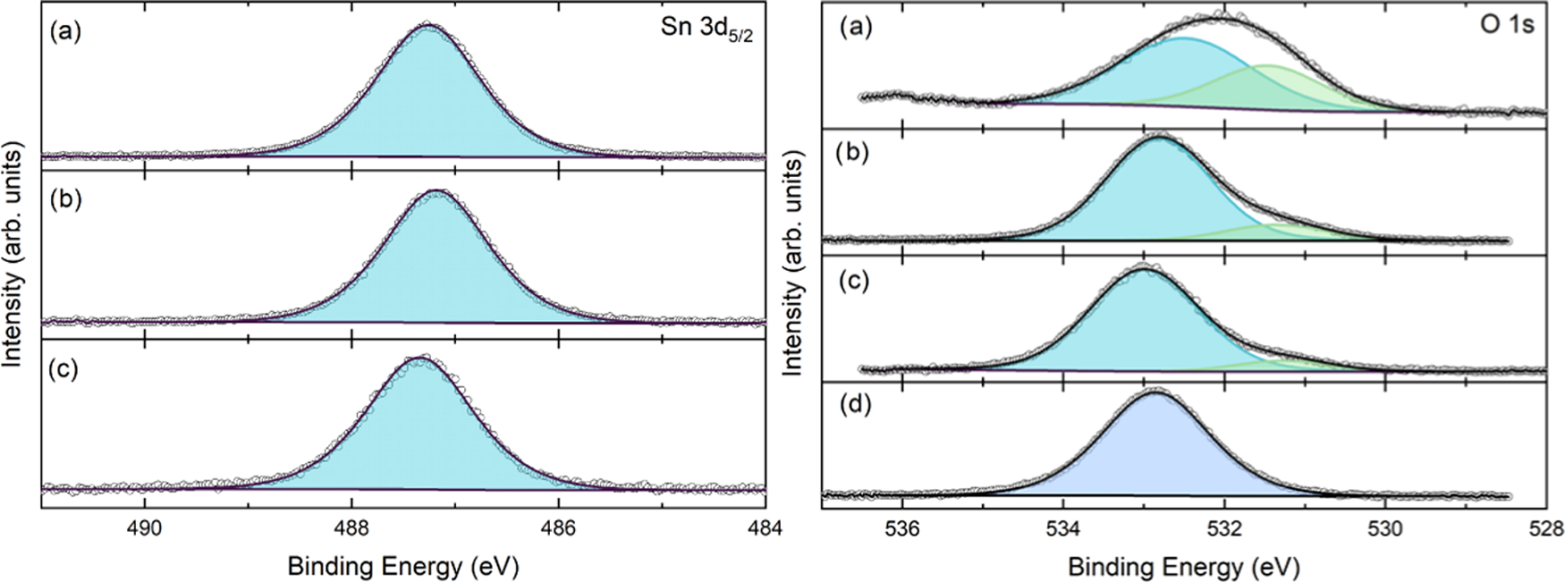
XPS spectra for Sn 3d (left) and O 1s (right) of SnO_2_-coated Au nanoparticles at different shell synthesis conditions: pH 3.5 and 60 °C with a precursor concentration of 15 mM after a reaction time of 180 min (a); pH 11.4 and 60 °C with a precursor concentration of 8.04 mM after a reaction time of 80 min (b); pH 11.4 and 80 °C with a precursor concentration of 1.13 mM after a reaction time of 5 min (c); and citrate-stabilized Au nanoparticles (d).

Figure 2 presents the XPS spectra of SnO_2_-coated gold nanoparticles synthesized at different conditions. According to the literature, commonly reported values for the binding energies (BE) and FWHM of Sn 3d_5/2_ and O 1s signals for non-intentionally doped SnO_2_ are 486.9 (1.04) and 530.8 (1.7) eV, respectively.^55−57^ Consistently, the results displayed in Figure 2 agree with the literature, confirming that the shell is exclusively composed of Sn^4+^ oxidized species (SnO_2_).^58^ The O 1s core-level position implies that the Fermi level (E_F_) is at the SnO_2_ conductionn band minimum.^57^ In contrast, the shift of the O 1s core-level emission of the sample synthesized at pH 3.5 can be explained by the intercalation of Na^+^ cations on the SnO_2_ shell as previously explained. Charge transfer from the SnO_2_ shell to the Au core is expected due to the large difference between the E_F_ of Au and that of SnO_2_,^31^ depending on the pH. During the shell growth around the Au cores, the charge transfer would induce a blue shift in the surface plasmon (SP) band position, as electrons are injected into the Au surface atoms.^31,48^ This fact may also explain the differences in the position of the SP band during shell syntheses (Figure S3) since the final position is the resultant from two, contrary, contributions: a red shift as the shell grows because of the change in the surrounding refractive index, and a blue shift due to the charge transfer from the SnO_2_ to Au surface.

Analysis of the PXRD patterns of SnO_2_ shows a crystallite size of ∼2.0 ± 0.5 nm for SnO_2_ shells obtained at pH 3.5, while the samples at pH 11.4 present a crystallite size of ∼5.6 ± 0.6 nm (Figure S8). A smaller crystallite size for the samples obtained at lower pH (3.5) reveals a lower crystallinity of the shell than for the samples at alkaline pH that could affect the growth of the shell. The lower crystallinity may also be affected by the Na^+^ doping that occurred at acid pH.

These results confirm the formation of SnO_2_ shells around the Au core and demonstrate that a high pH (11.4) is essential for the growth of the shell regardless of the temperature. A lower pH (3.5) leads to the formation of thin, Na^+^-doped SnO_2_ shells with pinholes, while at a higher pH (11.4), thicker, undoped SnO_2_ shells are obtained. The analysis highlights that slight changes in the synthesis protocol allow the design of SHINs targeted for each experiment, with pH 11.4 and 60 °C being the best option to obtain pinhole-free SnO_2_-coated Au NPs suitable for SHINERS.

### SnO_2_-Coated Au Nanoparticles for SHINERS

**Figure 3:**
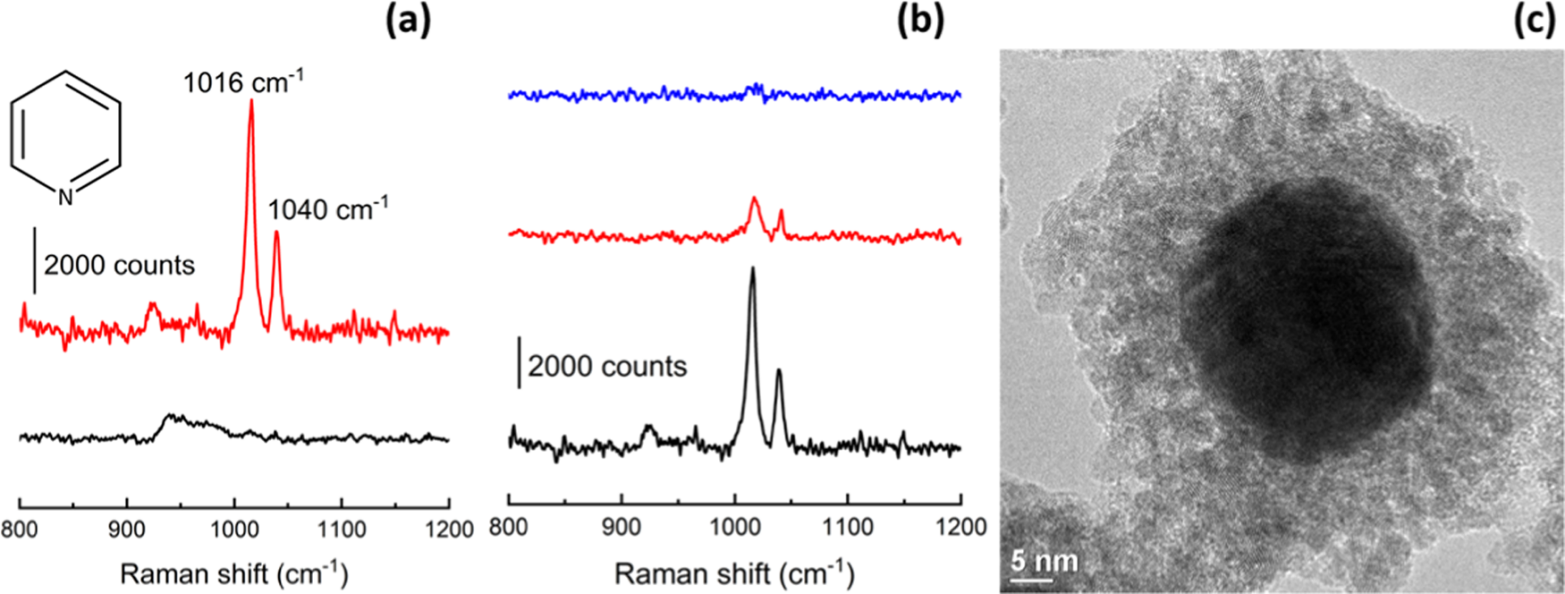
Raman signal of the pyridine molecule (top left) on SnO_2_-Au SHINs (pH 11.4 at 60 °C with a precursor concentration of 8.04 mM after 80 min reaction time) absorbed on Si (black line) and Au (red line) wafers (a). Enhancement test of SnO_2_-Au SHINs with 11 ± 3 nm (black line), 19 ± 4 nm (red line), and 24 ± 3nm (blue line) shell thicknesses measured by TEM (b). TEM image of a 11 ± 3 nm shell SnO_2_-Au SHIN (identical synthesis conditions as described for (a)) (c).

SnO_2_-coated Au NPs for use in SHINERS with highest enhancement were obtained at pH 11.4 and 60 °C with a precursor concentration of 8.04 mM after 80 min reaction time. The quality of the shells for use in SHINERS was studied by the pinhole test and magnitude of the enhancement in the Raman signal. For assessing these two properties, pyridine was employed as a probe molecule^10,59^ and the resultant spectra are summarized in Figure 3.

The adsorbed pyridine presents characteristic vibrational modes at 1016 and 1040 cm^–1^, corresponding to the ring breathing mode and asymmetric stretch, respectively.^17^ In Figure 3a, no Raman signal from the adsorbed pyridine is observed in the Raman spectrum of SHINs onto a Si wafer,^49,59^ while high-intensity pyridine bands appear in the Au wafer consistent with a pinhole-free shell suitable for SHINERS.^17^

As previously reported, the enhancement of the Raman signal depends on the distance between the Au core and the surface, so there is a direct relationship between the intensity of the Raman signal and the thickness of the shell (Figure S9).^13^ According to the literature,^16,59,60^ SiO_2_ shells greater than 10 nm exhibit no Raman enhancement at all. However, SnO_2_-coated Au NPs with a pinhole-free, 11 ± 3 nm shell offer a similar enhancement to SiO_2_-coated SHINs with a 3.2 ± 0.4 nm shell (Figures S10–S12), suitable to detect adsorbed species in electrode surfaces. Consequently, pinhole-free, functional SnO_2_-Au SHINs are obtained at 60 °C and show Raman signal increase with shells up to 19 ± 4 nm thick when the enhancement diminishes (Figure 3b). This fact notably would facilitate the synthesis process of the SHINs, since the range of shell thickness that allows an enhancement in SnO_2_-Au SHINs (11–19 nm) is greater than that for SiO_2_-coated SHINs (1–4 nm).

The physical origin for the increase in the Raman signal, especially when the shell is over 10 nm thick (Figure 3c), is not yet determined. The loss of the Raman signal intensity of pyridine (Figure 3b) when the shell thickness increases confirms a strong electromagnetic field gradient^60^ induced by the gold core on the SnO_2_-Au SHINs, but this is unlikely to explain all of the observed enhancement. Beyond the independent SERS effect of Au, an enlargement of the Raman signal attributed to charge transfer (CT) between the SnO_2_ conduction band orbital and an acceptor or donor molecular orbital of the adsorbed pyridine has also been reported.^61^ Nevertheless, the contribution of each type of enhancement is complex to determine, since other factors such as porosity of the shell and size of tin dioxide particles may play an important role in the optical properties of these materials.^62^

**Figure 4:**
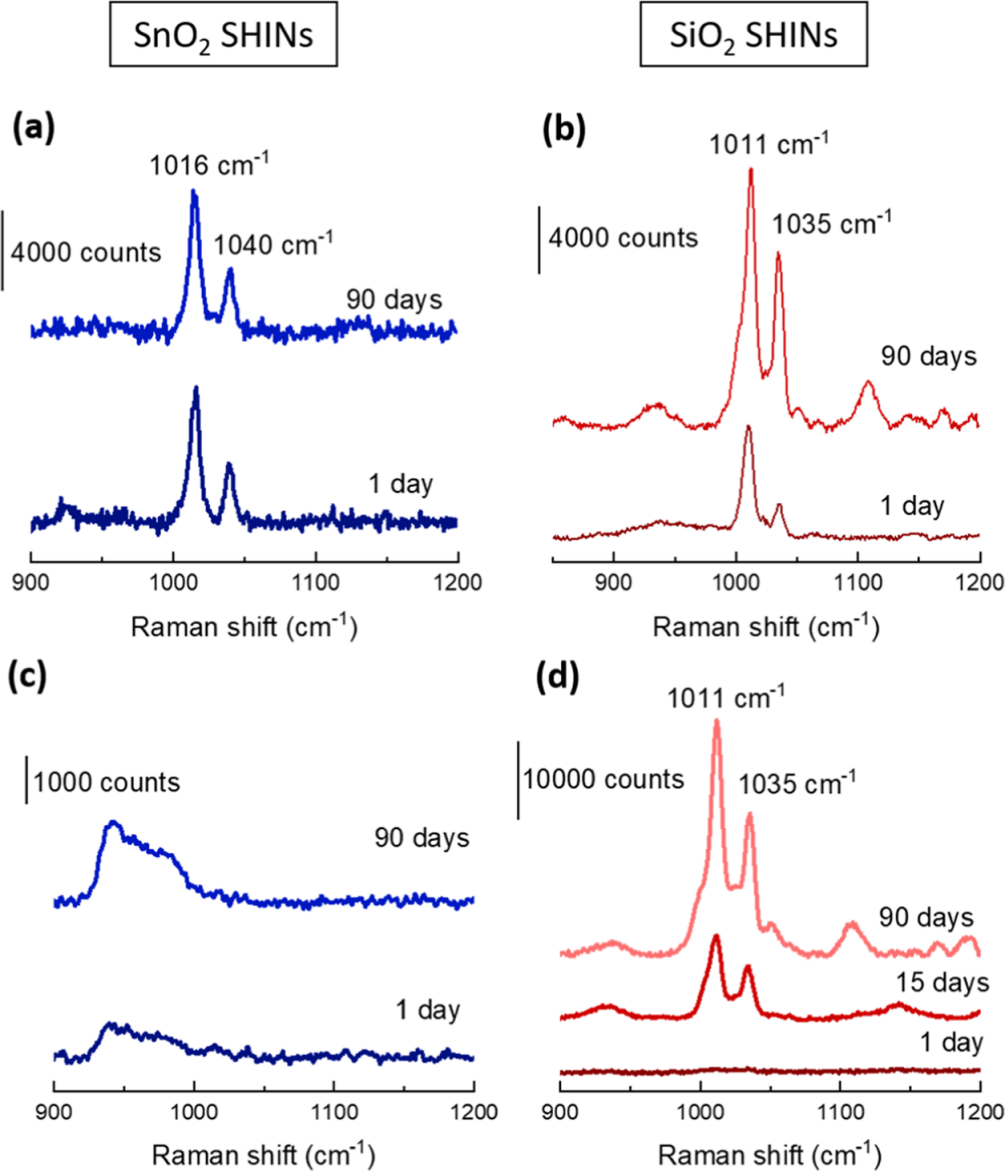
Comparison of the enhancement performance of SnO_2_ SHINs (a) vs SiO_2_ SHINs (b) and pinhole tests of SnO_2_ SHINs (c) and SiO_2_ SHINs (d) with a Raman laser wavelength of 785 nm.

The functional shelf life of SnO_2_-Au SHINs compared with SiO_2_-Au SHINs was studied under different storage conditions (Figure 4 and Figure S13). When the enhancement of the Raman signal is analyzed, no significant changes in the Raman signal intensity three months after the synthesis are observed for the deposited SnO_2_-Au SHINs (Figure 4a). An increase in the intensity of the Raman signal for SiO_2_-Au SHINs (Figure 4b) can be explained by the adsorption of pyridine onto the Au core after pinholes are formed on the shell. Moreover, while SnO_2_-Au SHINs presented no pinholes after 90 days (Figure 4c), SiO_2_-Au SHINs showed pinholes after 15 days (Figure 4d), indicating a greater durability for SnO_2_ shells compared to SiO_2_ coatings. Furthermore, a wider pH stability range for SnO_2_-Au SHINs is observed (pH 2–13) compared to the SiO_2_-Au SHINs (pH 4–11) (Figure S14), in agreement with the literature for SnO_2_ coatings in core/shell nanoparticles.^23^ While SiO_2_ shells dissolve at extreme pH, SnO_2_-Au SHINs present a full recovery of the SPR band upon pH neutralization when the negatively charged SnO_2_^–^ surface is recovered.^23^ These results represent a great advancement in the development of SHINs and imply, in addition to an increase in the applicability of SHINERS for alkaline and acidic systems, a greater ease for their synthesis and a lower frequency of synthesis, increasing the reproducibility of studied systems for academic purposes, and facilitating their wider use within the industry.

The increase in the Raman intensity of the bands at 1016 and 1040 cm^–1^ for SiO_2_ SHINs 3 months after their synthesis can be tentatively explained by direct contact of the pyridine with the Au core due to the pinholes on its surface. The rise in intensity of the pyridine bands is accompanied by the appearance of numerous additional bands at 930, 1128, 1160, and 1190 cm^–1^, which highlighted the degree of sensitivity to potential trace contamination once the pinholes form.

## Conclusions

Uniform, monodispersed, spherical, ca. 37 ± 5 nm Au NPs encased in SnO_2_ with a controlled shell thickness were prepared at pH 3.5 and 11.4, at 60 and 80 °C. The preparation of SnO_2_-coated Au NPs at pH 11.4 and 60 °C led to the formation of an approximately 11 nm thick, pinhole-free SnO_2_ shell, which was suitable for SHINERS. The SnO_2_-Au SHINs provided comparable Raman enhancement to the 3 nm thick SiO_2_ shells, due to the SnO_2_ SERS signal attributed to charge transfer and an SPR enhancement from the gold core. SnO_2_-Au SHINs were studied after three months and showed greater durability than SiO_2_-Au SHINs, exhibiting the same Raman enhancement as that of freshly prepared SnO_2_-Au SHINs and, importantly, without the presence of pinholes. Moreover, SnO_2_-Au SHINs offer higher pH stability (pH 2–13 vs pH 7–11.5 for SiO_2_ shells) and a longer lifetime than the widely used SiO_2_-Au SHINs, thus enabling the use of SHINERS within a wider range of electrochemical and catalytic environments.

## Supporting Information Available

TEM images of citrate-stabilized Au NPs and Au–SnO_2_ SHINs are given in the SI; pinhole and enhancement test results with both pyridine and rhodamine-6G are presented; UV–visible spectra, XPS and XRD results are shown for all different synthesis conditions used; enhancement of Au–SnO_2_ SHINERS under different storage conditions has been measured with Raman and results are displayed in the SI; pH stability of Au–SnO_2_ SHINERS is shown along with Au–SiO_2_ SHINERS (PDF)
