# Supplementary material for: Long-Life and pH-Stable SnO2-Coated Au Nanoparticles for SHINERS
Source: J Phys Chem C Nanomater Interfaces. 2022 Jul 13;126(29):12074–81. doi: 10.1021/acs.jpcc.2c02432 (PMC9340803; doi:10.1021/acs.jpcc.2c02432)
Supplement: Supplementary file 1 — jp2c02432_si_001.pdf [file jp2c02432_si_001.pdf]

Supporting Information

Long-Life And pH Stable SnO_2 Coated Au Nanoparticles For SHINERS

Julia Fernández-Vidal[1], Ana M. Gómez-Marín[2], Leanne A. H. Jones[3], Chih-Han Yen[1,4], Tim D. Veal[3], Vinod R. Dhanak[3], Chi-Chang Hu[4], Laurence J. Hardwick[1]*

[1] Stephenson Institute for Renewable Energy, Department of Chemistry, Peach Street, University of Liverpool, Liverpool, L69 7ZF, United Kingdom

[2] Department of Chemistry - Division of Fundamental Sciences (IEF) Aeronautics Institute of Technology (ITA) Praça Marechal Eduardo Gomes, 50 CEP 12228-900 | São José dos Campos/SP Brazil

[3]Stephenson Institute for Renewable Energy and Department of Physics, Peach Street, University of Liverpool, Liverpool, L69 7ZF, United Kingdom

[4] Department of Chemical Engineering, National Tsing Hua University, Hsinchu, 300044, Taiwan

*hardwick@liverpool.ac.uk

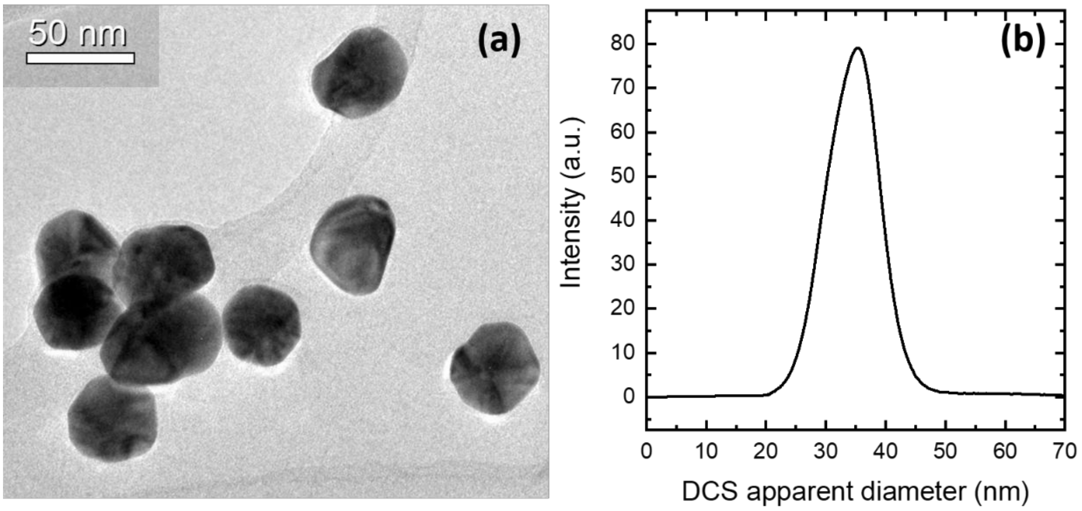

Figure S1. TEM image (a) and Differential Centrifugal Sedimentation (DCS) spectrum (b) of citrate-stabilized gold nanoparticles used for SHINs syntheses. TEM show them to be nearly spherical with an approximate diameter of ca. 43±9 nm. Similarly, DCS analysis showed an average size of 37±5 nm, considering the thin citrate layer adsorbed on Au surface. Au NPs size was estimated with DCS following the description in the literature for functionalized nanoparticles.[1,2]

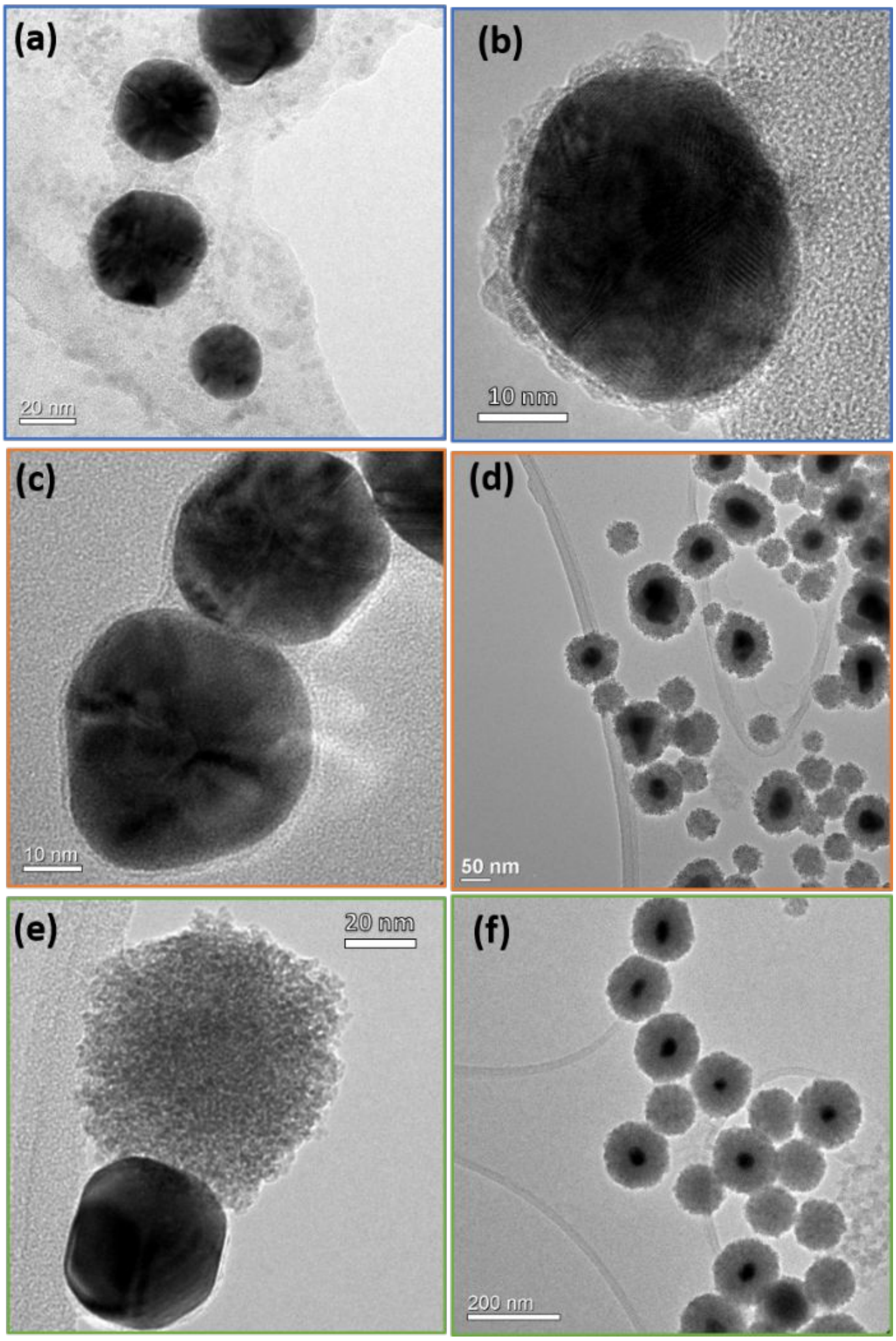

Figure S2. TEM images of SnO_2-coated SHINs. Syntheses at pH 3.5 and 60°C (-), pH 11.4 and 60°C (-), and pH 11.4 and 80°C (-) after 30 ((a), (c) and (e)), and 240 min ((b), (d) and (f)) reaction time. Na_2SnO_3 concentration details given in the Table 1 in the main paper. Figure S4c showed SHINs similar to SHINS described by Burgess et al.[3] However, the SHINs produced within this study exhibited pinholes at this stage and hence, were unsuitable for SHINERS measurements.

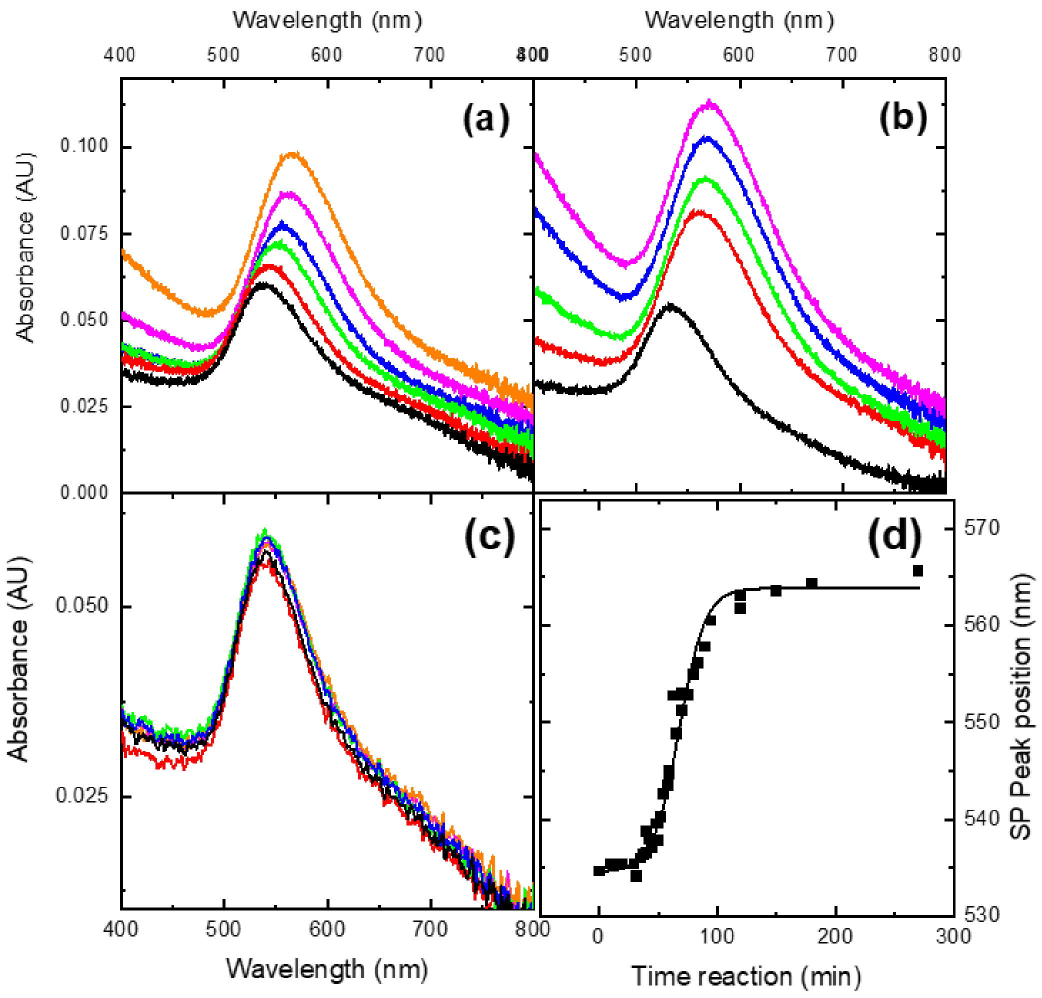

Figure S3. UV-Visible absorption bands of SnO_2-coated gold nanoparticles. Shell synthesis at pH 11.4 and 60°C with a precursor concentration of 8.04 mM after 10 (-), 60 (-), 70 (-), 90 (-), 150 (-) and 210 (-) minutes (a), at pH 11.4 and 80°C with a precursor concentration of 1.13 mM after 0 (-), 10 (-), 30 (-), 120 (-), 150 (-) minutes (b), and at pH 3.5 and 60°C with a precursor concentration of 15 mM after 0 (-), 30 (-), 60 (-), 120 (-), 150 (-), and 240 (-) minutes of reaction time (c). Sigmoidal tendency of the Surface Plasmon (SP) peak displacement vs time reaction for samples prepared at pH 11.4 and 60ºC (d).

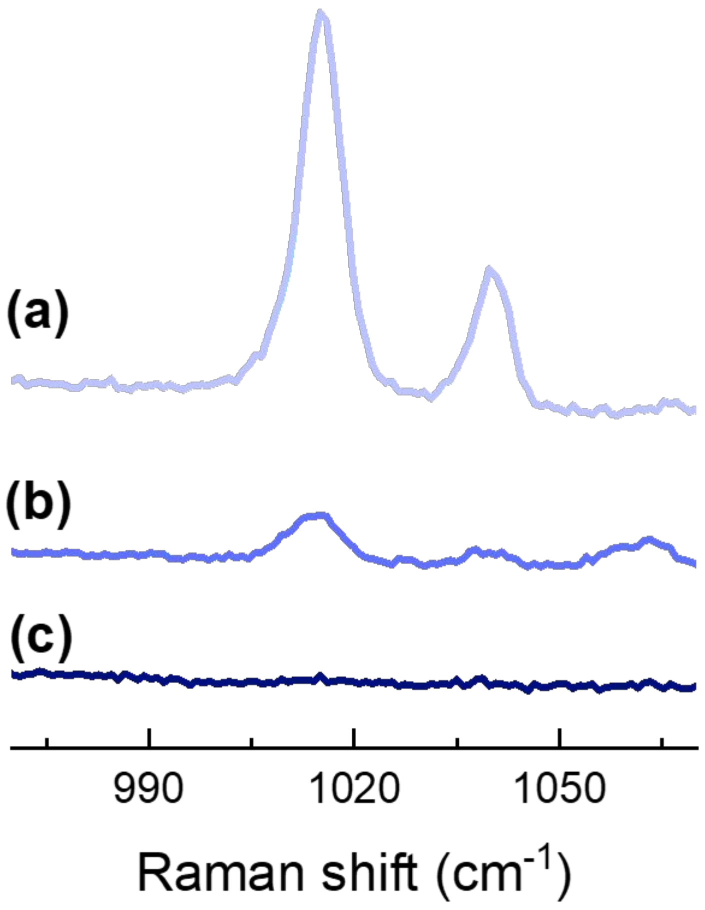

Figure S4. Raman signal of pyridine molecule on Si wafer with SnO_2-coated SHINs synthesized at (a) pH 3.5 and 60ºC, (b) pH 11.4 and 80ºC and (c) pH 11.4 and 60ºC for pinhole tests after 240 min reaction time. Only shells prepared at pH 11.4 and 60ºC present no pinholes, suitable for SHINERS.

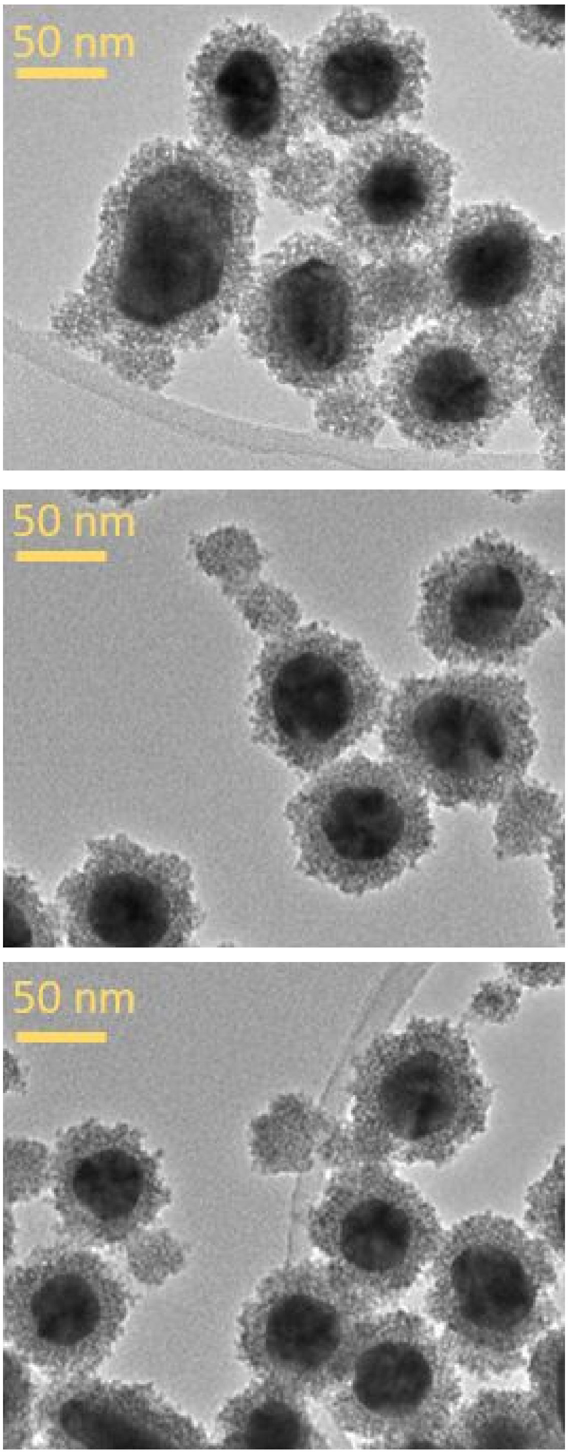

Figure S5. TEM images of Au-SnO_2 SHINs at pH 11.4 and 60 ºC at various locations with a precursor concentration of 8.04 mM after reaction time 80 min and Na_2SnO_3 concentration of 8.04 mM. TEM show the cores to be nearly spherical with an approximate diameter of ca. 43±9 nm in agreement with DCS data (Figure S1) and a shell thickness of 11±3 nm.

Nanoparticles size variation are within the expected polydispersity for the experimental procedure.[4] In addition to the SnO_2 that appears surrounding the gold nanoparticles, spherical structures of this material are observed in TEM images (also observed in Figure S2), especially at higher reaction times and temperatures. The presence of species that absorb at different wavelengths (SnO_2 and Au) can also contribute to intermediate absorption spectra as discussed in the main paper.

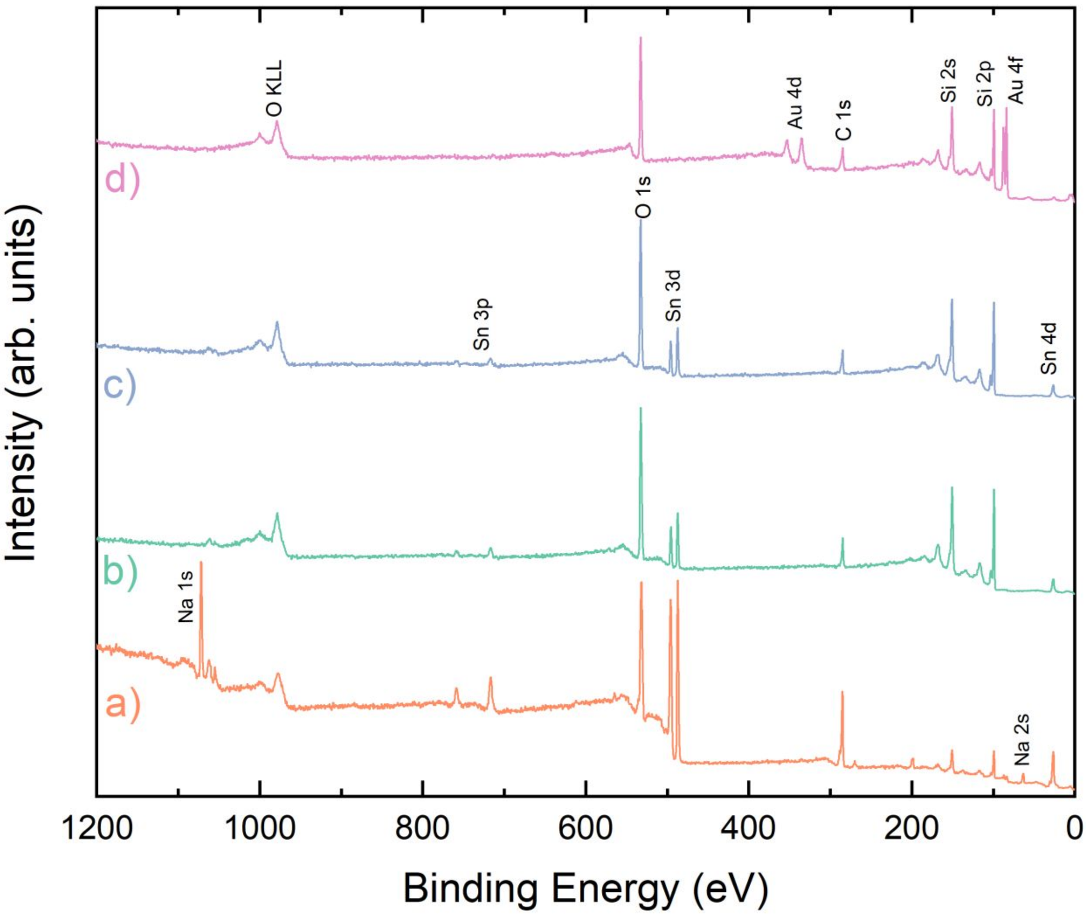

Figure S6. XPS survey spectra of SnO_2 coated Au nanoparticles at different shell syntheses conditions. pH 3.5 and 60 ºC with a precursor concentration of 15 mM after reaction time 180 min (a); pH 11.4 and 60 ºC with a precursor concentration of 8.04 mM after reaction time 80 min (b); pH 11.4 and 80 ºC with a precursor concentration of 1.13 mM after reaction time 5 minutes (c); and citrate stabilized AuNPs (d). Si 2s and 2p peaks are expected and come from the Si wafer used to deposit the SHINs.

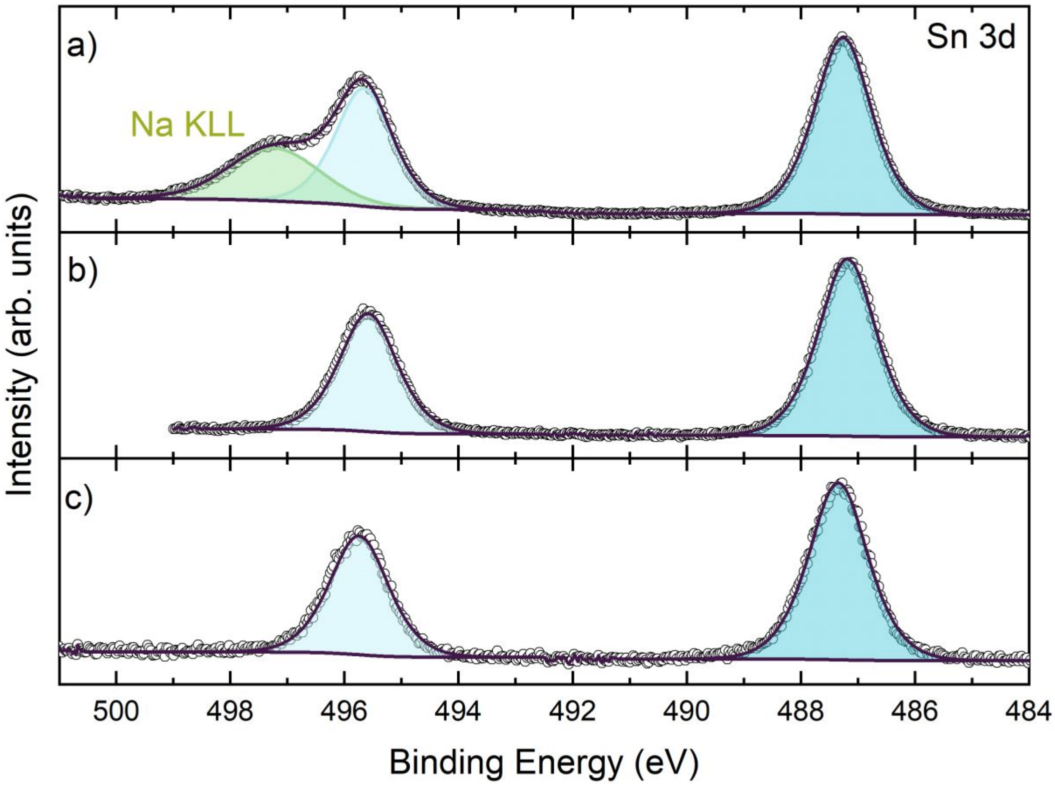

Figure S7. XPS spectra for Sn 3d of SnO_2 coated Au nanoparticles at different shell synthesis conditions. pH 3.5 and 60 ºC with a precursor concentration of 15 mM after reaction time 180 min (a); pH 11.4 and 60 ºC with a precursor concentration of 8.04 mM after reaction time 80 min (b); pH 11.4 and 80 ºC with a precursor concentration of 1.13 mM after reaction time 5 minutes (c).

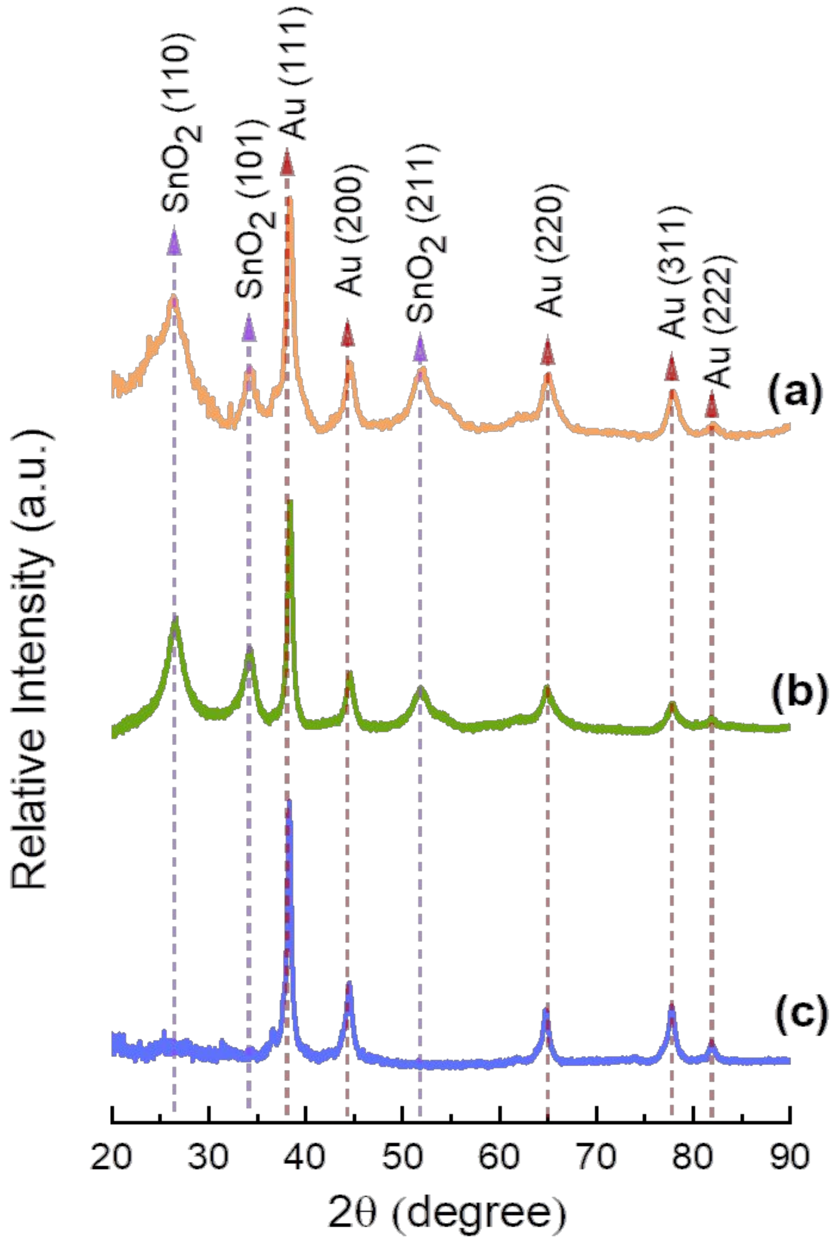

Figure S8. Powder X-Ray Diffraction (PXRD) patters of Au-SnO_2 SHINs. Shell syntheses at pH 11.4, 60°C with a precursor concentration of 8.04 mM after reaction time 180 min (a), 80°C with a precursor concentration of 1.13 mM after reaction time 120 min (b), and at pH 3.5, 60°C with a precursor concentration of 15.0 mM after reaction time 180 min (c).

Information about composition and crystalline structure of Au-SnO_2 SHINs was obtained by PXRD measurements. Samples described in Table 1 in the main text were used for PXRD analysis due to its thicker shell. Distinctive peaks for Au NPs with a face-centred cubic (fcc) structure are observed (JCPDS 89-3697), with a marked contribution of (111) planes. Furthermore, data in Figure S11 confirm the synthesis of SnO_2 shells of crystalline cassiterite structure (JCPDS 41-1445) on Au cores, as expected from thermodynamic considerations since SnO_2 cassiterite is the stable phase precipitated from solutions at pH<~11.7.[5] No other particular peaks were found, suggesting that the level of crystalline impurities, other than SnO_2 phases, is lower than the detection limit of the diffractometer (< 5%). Shifts of c.a. ±0.1° in the SnO_2 XRD peak positions towards a higher 2θ value relative to the positions of the tetragonal SnO_2 structure (JCPDS 41-145) are found, and are especially evident for samples synthesized at 80°C (Figure S11b). This shift may be ascribed to the presence of residual stress and microstrains induced by defects in the SnO_2 shell, i.e. systematic stacking and twin faults, point defects, among others.[6,7] Contrarily, regardless of the shell synthesis conditions, no peak shift is observed for Au, confirming that the Au lattice structure is not modified during this process. Calculated crystallite size of Au core of NPs synthesized at pH 11.4 and 60°C is ~9 ± 1 nm, while a value of ~12 ± 2 nm was found for the other two samples, suggesting a similar crystallite (within the margin of error).[8] For SnO_2 shells, also a similar crystallite size of ~5.6 ± 0.6 nm was calculated for samples coated at high pH, while a smaller size of ~2.0 ± 0.5 nm was approximated for the synthesis at low pH (3.5). Wider and smaller peaks found in XRD peaks attributed to SnO_2 reveal a low crystallinity of the shell, and the possible existence of other amorphous complex phases[9] of samples synthesized at pH 3.5.

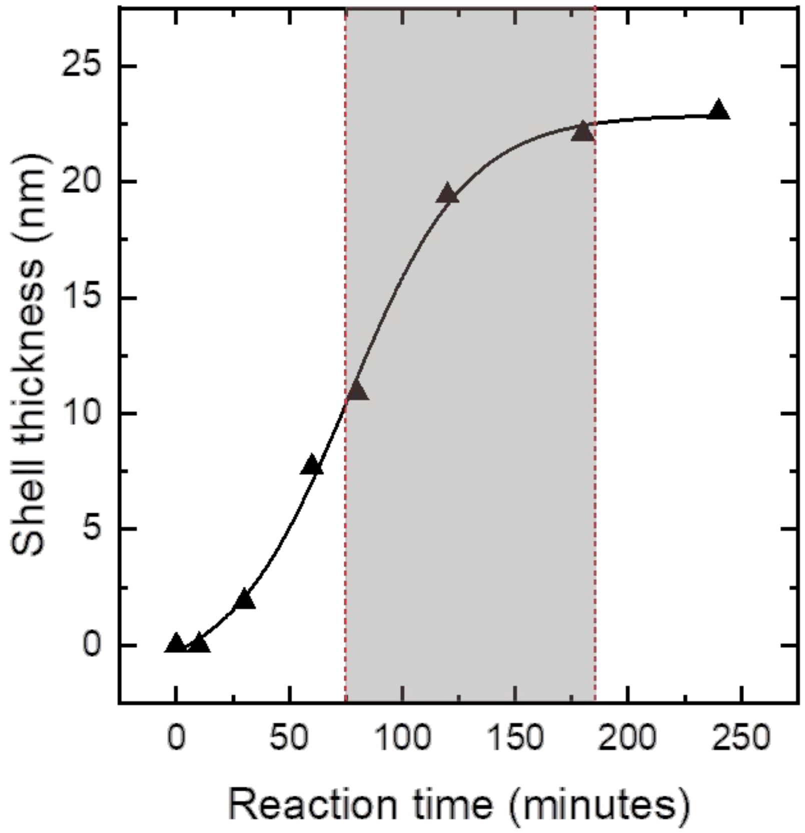

Figure S9. Sigmoidal tendency of the shell thickness (measured via TEM) vs time reaction for samples prepared at pH 11.4 and 60ºC. The growth of the shell presents a sigmoidal tendency typical for nucleation processes where the fastest growth occurs between 25-150 minutes. Red region represents the pinhole-free samples which also present enhancement making them suitable for SHINERS. Samples above 180 min time reaction present no enhancement and samples taken below 75 min show pinholes. High enhancement in samples with pinholes is explained due to the adsorption of pyridine onto the Au core surface.

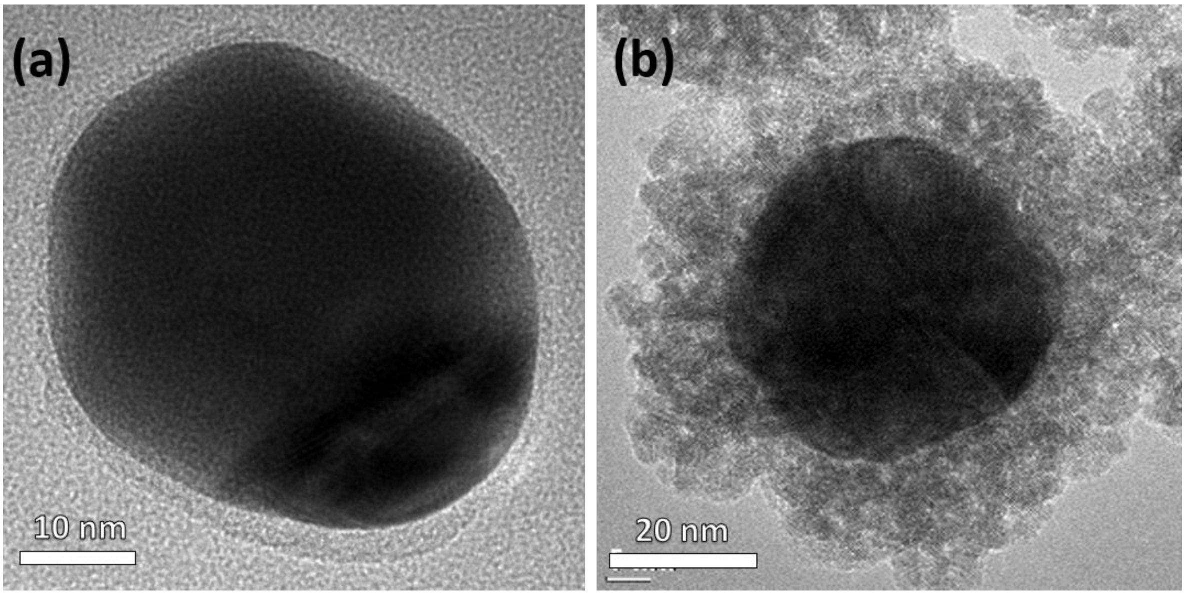

Figure S10. TEM images of an (a) Au-SiO_2 SHIN and an (b) Au-SnO_2 SHIN suitable for SHINERS.

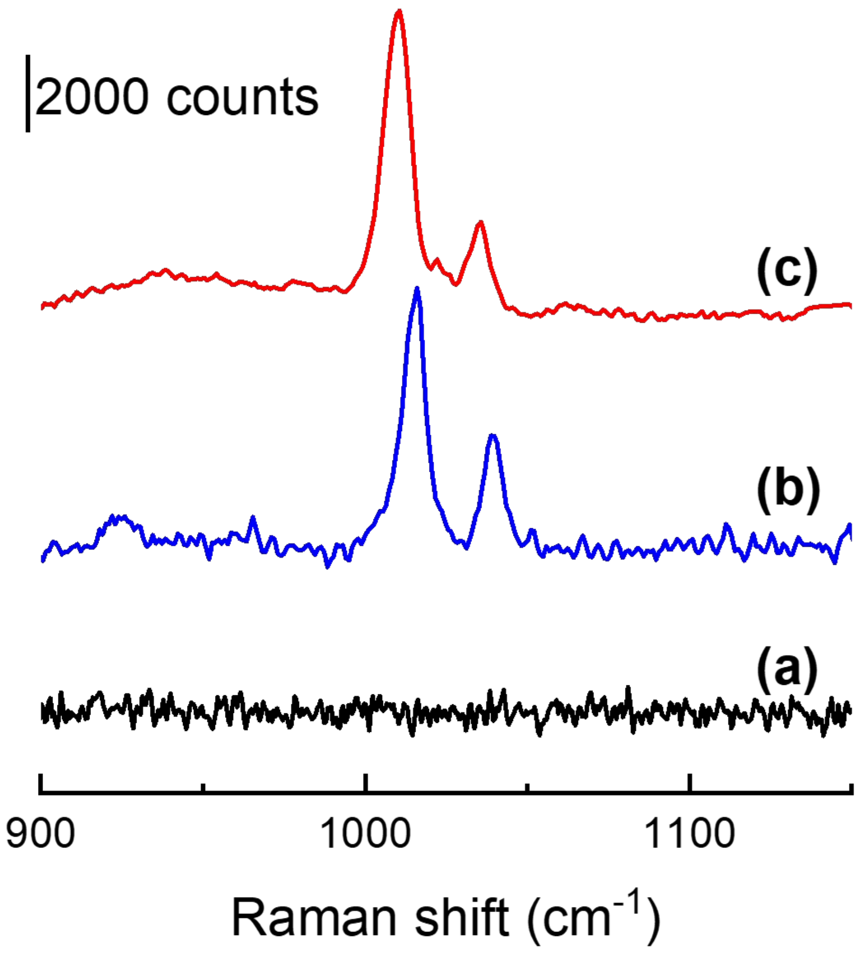

Figure S11. Raman signal of pyridine molecule on Au wafer without SHINs (a) and with (b) SnO_2-coated SHINERS and (c) SiO_2-coated SHINERS. The ca. 4-5 cm^{-1} wavenumber shift of both the measured absorbed pyridine bands can be observed.

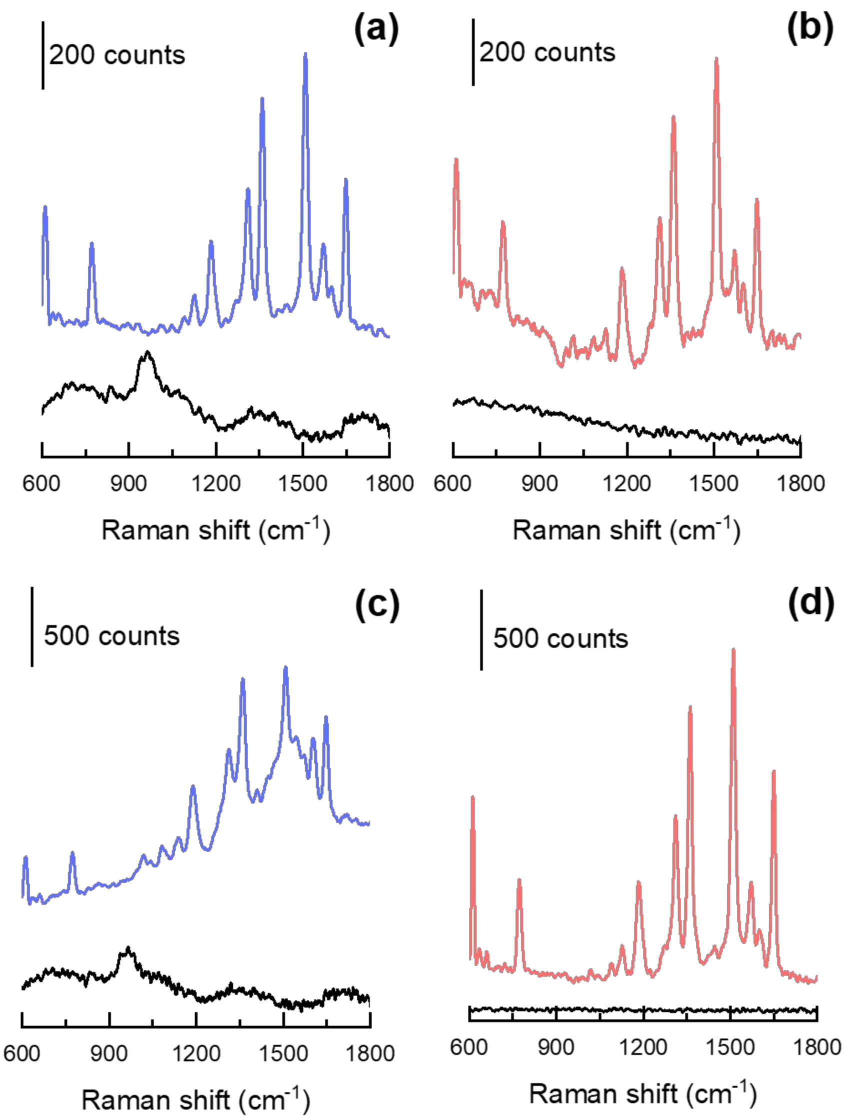

Figure S12. (a) Raman signal of rhodamine-6G molecule absorbed on Si wafer with (-), and without (-) SnO_2-coated SHINERS (pH 11.4 60 ºC with a precursor concentration of 8.04 mM after 80 min reaction time) measured with a 785 nm laser. (b) Raman signal of rhodamine-6G molecule absorbed on Au wafer with (-), and without (-) SnO_2-coated SHINERS measured with a 785 nm laser. (c) Raman signal of rhodamine-6G molecule absorbed on Si wafer with (-), and without (-) SnO_2-coated SHINERS (pH 11.4 60 ºC with a precursor concentration of 8.04 mM after 80 min reaction time) measured with a 633 nm laser. (d) Raman signal of rhodamine-6G molecule absorbed on Au wafer with (-), and without (-) SnO_2-coated SHINERS measured with a 633 nm laser.

Enhancement tests represented in **Figure S12** were taken via drop casting 5 μL of 0.1 mM rhodamine 6G solution and the measurements were taken both with 633 nm and 785 nm laser excitation. Exposure time of the laser was 10 seconds using a 0.1% filter. Rhodamine 6G has previously been used to study the enhancement of the SnO_2-coated SHINERS since it absorbs on both metallic and non-metallic substrates.[10] As represented in **Figure S12**, no Raman signal is observed for rhodamine 6G in either Au or Si wafers, while a large enhancement occur when SnO_2 SHINERS are present. These results highlight that the origin of enhancement of the Raman signal originates from the deposited SHINERS.

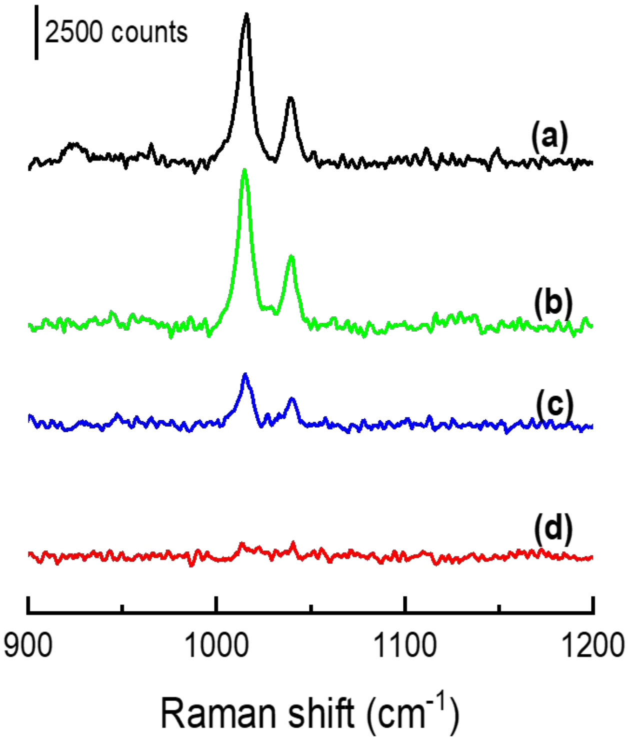

Figure S13. Stability and lifetime of functional SHINs after their synthesis were checked by storing them under different conditions after 1 day (a) and after 3 months, (b) deposited on substrate, (c) in aqueous suspension, and (d) as a precipitate after centrifugation. Three months after the synthesis, the SHINs that were deposited presented no-pinholes and an enhancement of the same intensity (b) as that of the nanoparticles one day after their coating. While SiO_2-coated SHINs show stability for approximately 10 days in aqueous solution before aggregating (Figure 4 in the main text), SnO_2-coated SHINs do not show any signs of aggregation in any of the storage conditions. Even though neither storage method presented pinholes, the enhancement was diminished by a factor of 2.7 (calculated from the integrated asymmetric stretch band of pyridine) for the SHINs in aqueous solution (c) and disappeared for those that were in the precipitate (d). These results indicate that even so, in general, SnO_2 shells have a greater stability than SiO_2 shells, the storing condition is an important factor in the durability of the SHINs, being the deposition of them preferable to maintain the enhancement of the SHINs.

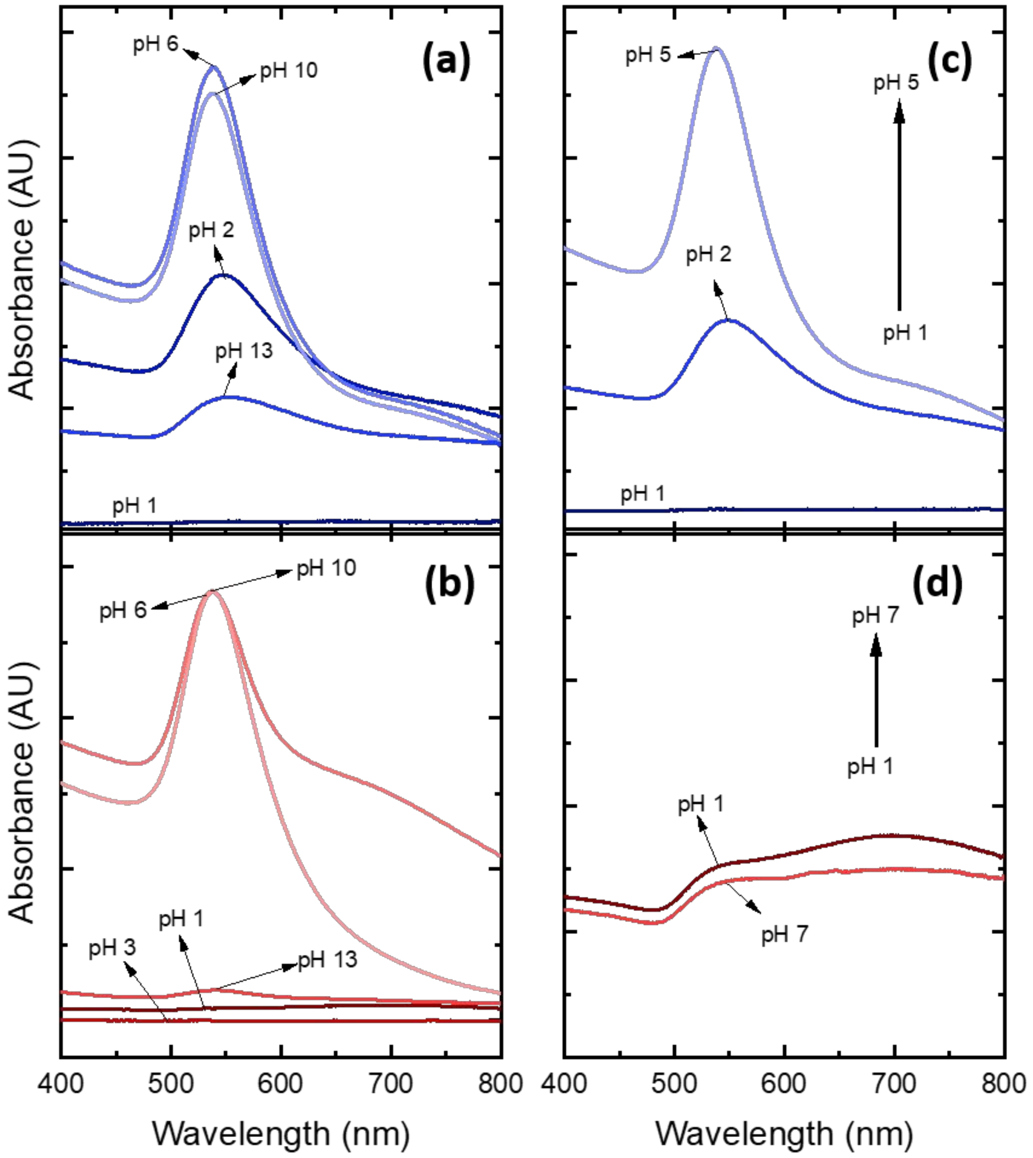

Figure S14. UV-Visible absorption spectra of Au-SnO_2 SHINs (a) and Au-SiO_2 SHINs (b) 8 days after changing the pH. UV-Visible absorption spectra of dispersed Au-SnO_2 SHINs (c) and Au-SiO_2 SHINs (d) at pH 1 and recovery of the SP band. SnO_2 shells have a greater robustness and pH stability than SiO_2 shells. Additionally, Au-SnO_2 SHINs showed to recover the SP band at pH 5 after exposing them to pH 1.

References

(1) Davidson, A. M.; Brust, M.; Cooper, D. L.; Volk, M. Sensitive Analysis of Protein Adsorption to Colloidal Gold by Differential Centrifugal Sedimentation. *Anal. Chem.* **2017**, *89* (12), 6807–6814.

(2) Krpetić, Ž.; Davidson, A. M.; Volk, M.; Lévy, R.; Brust, M.; Cooper, D. L. High-Resolution Sizing of Monolayer-Protected Gold Clusters by Differential Centrifugal Sedimentation. *ACS Nano* **2013**, *7* (10), 8881–8890.

(3) Barlow, B. C.; Guo, B.; Situm, A.; Grosvenor, A. P.; Burgess, I. J. Shell Isolated Nanoparticle Enhanced Raman Spectroscopy (SHINERS) Studies of Steel Surface Corrosion. *J. Electroanal. Chem.* **2019**, *7*, 410.

(4) Haiss, W.; Thanh, N. T. K.; Aveyard, J.; Fernig, D. G. Determination of Size and Concentration of Gold Nanoparticles from UV−Vis Spectra. *Anal. Chem.* **2007**, *79* (11), 4215–4221.

(5) Ingham, B.; Toney, M. F. X-Ray Diffraction for Characterizing Metallic Films. In *Metallic Films for Electronic, Optical and Magnetic Applications: Structure, Processing and Properties*; 2013.

(6) Rai, D.; Yui, M.; Schaef, H. T.; Kitamura, A. Thermodynamic Model for SnO_2(Cr) and SnO_2(Am) Solubility in the Aqueous Na^+-H^+-OH^--Cl^--H_2O System. *J. Solution Chem.* **2011**, *40* (7), 1155–1172.

(7) Cullity, B. D. Elements of X-Ray Diffraction, 2nd Edition. *Addison-Wesley Publ. Co. Read. MA* **1978**, 100-105,277-279.

(8) Scherrer, P. Bestimmung Der Größe Und Der Inneren Struktur von Kolloidteilchen Mittels Röntgenstrahlen. *Nachrichten von der Gesellschaft der Wissenschaften zu Göttingen, Math. Klasse* **1918**.

(9) Ravaro, L. P.; De Andrade Scalvi, L. V. Influence of PH of Colloidal Suspension on the Electrical Conductivity of SnO_2 Thin Films Deposited via Sol-Gel-Dip-Coating. *Mater. Res.* **2011**, *14* (1), 113–117.

(10) Galloway, T. A.; Cabo-Fernandez, L.; Aldous, I. M.; Braga, F.; Hardwick, L. J. Shell Isolated Nanoparticles for Enhanced Raman Spectroscopy Studies in Lithium-Oxygen Cells. *Faraday Discuss.* **2017**, *205*, 469–490.
